# Supplementary material for: Effectiveness and Safety of Ayurvedic Medicines in Type 2 Diabetes Mellitus Management: A Systematic Review and Meta-Analysis
Source: Front Pharmacol. 2022 Jun 8;13:821810. doi: 10.3389/fphar.2022.821810 (PMC9213670; doi:10.3389/fphar.2022.821810)
Supplement: Supplementary file 1 [file DataSheet1.zip › Supplementary Material/Forest and Funnel Plots/Trigonella foenum-graecum/HDL-C.pdf]

Trigonella foenum-graecum - HDL-C

Study	Intervention N	Intervention Mean	Intervention SD	Comparator N	Comparator Mean	Comparator SD	Mean Diff. with 95% CI	Weight (%)
Gupta 2001	12	47.20	12.90	13	44.15	17.10	3.05 [-8.90, 15.00]	8.80
Rafraf 2014	44	32.72	15.84	44	41.65	17.67	-8.93 [-15.94, -1.92]	14.30
Singh 2016	20	43.06	3.92	20	43.44	5.24	-0.38 [-3.25, 2.49]	19.66
Gholaman 2018	10	48.20	5.30	10	39.10	3.34	9.10 [5.22, 12.98]	18.50
Kandhare 2018	48	47.40	8.99	53	45.53	7.74	1.87 [-1.39, 5.13]	19.23
Rashid 2019	32	44.08	7.35	32	39.83	4.64	4.25 [1.24, 7.26]	19.51
**Overall**							1.79 [-2.84, 6.42]	

Heterogeneity: $\tau^2 = 26.28$, $I^2 = 86.69\%$, $H^2 = 7.51$

Test of $\theta_i = \theta_j$: $Q(5) = 26.63$, $p = 0.00$

Test of $\theta = 0$: $z = 0.76$, $p = 0.45$

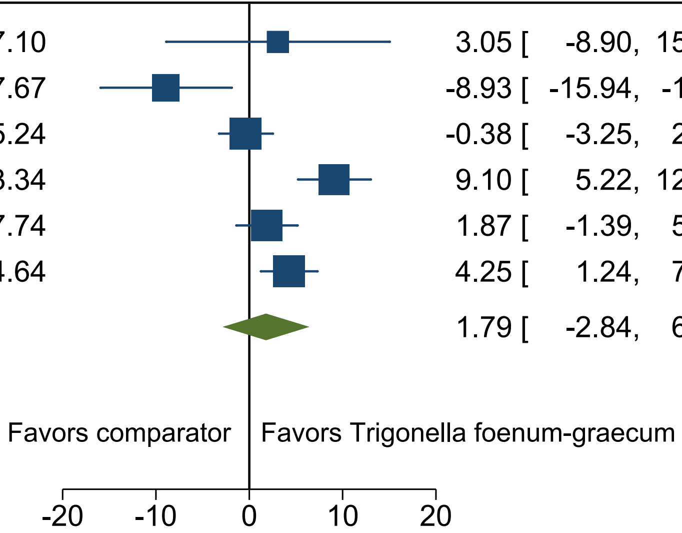

Random-effects REML model
